# A dynamical neural simulation mapping feature-based attention to location with non-linear cortical circuits

**DOI:** 10.1186/1471-2202-12-S1-P55

**Published:** 2011-07-18

**Authors:** David G Harrison, Marc de Kamps

**Affiliations:** 1 School of Computing, University of Leeds, Leeds, West Yorkshire, LS2 9JT, UK

## 

Visual attention mechanisms allow the visual cortex to direct cortical processing to salient locations within a visual scene, at the expense of diminished processing of stimuli at other locations. The deployment of attention to a given location is known as spatial attention. Attention may also be applied to a known feature in a visual scene, such as a colour or shape, enhancing its occurrences across the visual field. In [1], a model of binding distributed feature representations through feature-based attention predicted that attentional feedback would act in all positions of the visual scene in low level visual areas. There is now experimental evidence that this mechanism exists [2].

We present here a neural dynamical model of feature-based attention that directs attention to salient locations in a multi-object scene through interaction of stimulus driven and cue driven neural activation. This interaction is managed by a disinhibition circuit gating output to a retinotopic neural region of the dorsal stream, the lateral intraparietal area (LIP). When visual stimuli are presented to primary visual cortex (V1), neural activation spreads into higher ventral stream areas through increasing neural receptive fields between layers. Stimulus driven neurons activate excitatory output and inhibitory interneuron populations in the disinhbition circuit. When a salient feature is cued by activation of its associated anterior inferotemporal (AIT) neuronal population, neural activity propagates to lower visual areas across all locations of the visual field. Activity in this top-down flow inhibits the inhibitory interneuron population allowing excitatory output from the disinhibition circuit to LIP in a retinotopic manner where activity is correlated in the stimulus and cue driven pathways. In areas where the two pathways do not match, local inhibition in the disinhibition layer prevents neighbouring circuits' excitatory populations from outputting to LIP.

The model exhibits effects of feature-based attention, such as the feature similarity gain principle and biased competition through interactions of stimulus and cue initiated neural activity. The dynamical nature of the model as neural populations of Wilson-Cowan differential equations allows the time-course of neural activity to be investigated.

## Conclusions

Although originally designed as a model for binding, the model replicates the experimental correlates of feature-based attention, which strongly suggests its functional role is actually binding An important prediction of the model is that local non-matches between cue and stimulus are the most important information projected from the ventral to the dorsal stream and that this interaction is highly non-linear. We present candidates for neural circuits implementing this interaction.
